# Limits of a Second Language: Native and Second Languages in Management Team Communication

**DOI:** 10.3389/fpsyg.2021.580946

**Published:** 2021-09-21

**Authors:** Jan Ketil Arnulf, Wanwen Dai, Hui Lu, Zhe Niu

**Affiliations:** ^1^BI Norwegian Business School, Oslo, Norway; ^2^Department of Marketing, Business School of Nanjing University, Nanjing, China

**Keywords:** second language speakers, management teams, communication, China, meta-cognition, international organizations

## Abstract

Cultural differences in speech acts are common challenges in management involving Chinese and Western managers. Comparing four groups – Native-speaking Chinese, English-speaking Chinese, Chinese-speaking Westerners, and non-Chinese- speaking Westerners, we assessed the effects of language and ethnicity on the ability to predict communication obstacles in a management team scenario. Bilingual subjects were less likely to be influenced by ethnic biases. Still, bilinguals were not more likely to adjust their metacognitions about communication toward those of the native speakers. The study creates a link between management, cognition and linguistics, as well as having consequences for the study of metacognition in cross-cultural management.

## Introduction

Communication plays a crucial role in management (Mintzberg, 2009), and it is even seen as what organization is “about” (Weick, 1995). The most obvious culturally dependent obstacle to cross-cultural management is language differences, making the ability to speak several languages a necessity and an adaptive advantage (Peltokorpi, 2008; Zhang and Peltokorpi, 2016) but even people speak the same language at work, the use of second language as communication tool may have unforeseen effects (Tenzer et al., 2014). How does the native language of managers influence the way information is exchanged in a management team? And how far will speaking a foreign language enable a manager to anticipate the reactions of foreign co-workers?

The aim of this study is to explore how differences in managerial communication may be influenced by cognitive structures related to language, but on a more profound level than what is covered by simply learning a foreign language (Henrich et al., 2010; Jentjens, 2021). As will be argued below, different native languages may predispose people to different communicational habits with consequences for how verbal interaction shapes interaction among organizational participants. The ability to understand these differences and have meta-cognitive perceptions of them may not develop until the speakers acquire almost native levels of mastery in the foreign language, and thus still cause misunderstandings among people who have business-level understanding of each other’s language (Wang et al., 2014; Beeler and Lecomte, 2017). This field of study is an important borderline between native and corporate cultures because most cultural obstacles need to be sorted out in verbal interaction for organizations to overcome them (Wilkinson et al., 2005; Pavlenko, 2016).

The idea that one’s native language shapes the speakers’ world view and their communication habits has been subject of controversy for hundreds of years and most famously in social sciences as the Sapir-Whorf-hypothesis (Whorf, 1944, 1956; Gumperz and Levinson, 1996). After decades of controversy around linguistic relativity, the linguistic relativity hypothesis and a somewhat weaker linguistic determinism principle seems to be supported by a growing number of studies (Lucy, 1996; Boroditsky, 2001, 2011; Papafragou et al., 2002; Fausey et al., 2010; Imai et al., 2010). The worldview imposed by one’s native language is a source of ethnocentrism as humans have a tendency to assume invariance in their cognitive structures and communicative principles. Because of this, there is a tendency for second language speakers to transfer the cognitive and communicative habits from their native language into the newly acquired speaking arena. This can create types of miscommunications that are sometimes obvious, but at other times can go undetected or create barriers that are unforeseen since both parties are under the impression of speaking the same language (Gumperz, 1996; Enfield, 2007).

The research question of this study is to explore how native speakers of two widely different languages, i.e., English and Chinese, are able to predict a commonly occurring type of communication barrier in cross-cultural management. We then proceed to investigate how the ability to speak these languages as second language may improve these speakers’ ability to understand and predict the communication obstacles in the same situation. Finally, we look at these speakers’ meta-cognitive grasp of the problem they face.

This study contributes to research on cross-cultural management communication and to the issue of linguistic relativity by combining these perspectives in the exploration of a commonly encountered communicative obstacle in global management. It also offers explanations for differences in managerial behaviors that may be of practical value in cross-cultural training.

## Theory

The motivation for this study is grounded in two recently emerging strands of research on the psychology of cross-cultural management. Primarily, a growing body of research on indigenous theories of leadership and management has exposed the limitations of imposing constructs from one part of the world to another (Li, 2012). For example, a recent study of cross-cultural management team research found a series of methodological paradoxes that can only be adequately addressed by taking the behavioral implications of abstract constructs into account (Casas Klett and Arnulf, 2020).

Secondarily, the growing use of digital text algorithms (see Arnulf and Larsen, 2021) has documented that the prevalent use of Likert-scale measurement methods may be unable to detect true cultural differences (Arnulf and Larsen, 2020). The implication of these two lines of research point in the same direction: To capture the cultural differences that also make a difference in cross-cultural management, it is important to address how verbal behavior is connected with actual patterns of communications and the ensuing behavioral enactments of organizational communication. For this reason, the present study is addressing how different ethnic origins will influence communicative behavior and understanding of a paradigmatic case in managerial communication.

Being at the opposite ends of the individualism-collectivism spectrum, Eastern and Western cultures often show wide differences (Toffoli and Laroche, 2002; Casas Klett and Arnulf, 2020). A review by Schmitt et al. (1994) found a series of differences affecting management and business such as face saving, time perspective and differences in individualism/collectivism (Hofstede, 1980; Hofstede et al., 2010). Cultural differences have also been found to affect managerial decision making (Ralston et al., 1999; Casas Klett and Arnulf, 2020).

Specifically, the different management communication styles of Chinese and Western managers have been the subject of numerous studies (Wilkinson et al., 2005; Li, 2012; Ma and Tsui, 2015; Lin et al., 2018; Wong et al., 2018; Casas Klett and Arnulf, 2020). A common situation causing frequent misunderstandings and practical challenges in cross-cultural organizations and business is when Westerners approach a situation by inviting participants to “speak up” while East Asians may remain indirect, unresponsive or even totally silent. Westerners are commonly more direct and talkative in their communication style, while East Asians are often less outspoken, more restricted and more indirect in their interaction with others (Chen and Tjosvold, 2006). This may create situations where Westerners ask questions that are seemingly not answered at all, or where the answers are perceived as uninformative, leaving the Westerners uncertain about how the interaction will proceed. Situations like these are prototypical as examples of the widely different conceptions of “dialogue” between East and West, and are of considerable practical and even financial value to cross-culturally operating organizations (Wilkinson et al., 2005).

There are many kinds of situations where this problem may arise (Arnulf and Kristoffersen, 2014). For the present study, we have focused on one specific situation in which a Western expat CEO who has newly arrived in China tries to stimulate his top management team to an open exchange of viewpoints and ideas about the company’s present challenges. Where the Westerner wants to be inclusive and stimulate a participating and empowering style, the Chinese members of the management team do not want to voice their opinions. The meeting therefore ends on a somewhat awkward tone and with all participants feeling frustrated about the process (Arnulf, 2014).

To open up and help untangle this situation, we believe it is helpful to combine theories on the psychology of language with theories on culturally dependent cognition to explore and explain the ensuing differences in the psychology of cross-cultural management. The problematic role of language in shared social reality was succinctly described by Whorf (1956, p. 271): “Whenever agreement or assent is arrived at in human affairs, and whether or not mathematics or other specialized symbolisms are made part of the procedure, this agreement is reached by linguistic processes, or else it is not reached.”

The situation we are exploring is one where all participants have linguistic competences to understand the actual sentences being spoken, but where their implications are mutually confusing to the participants. Moreover, they do not have the meta-cognitive skills to address and solve the stalled situation. In what follows, we will draw on various lines of research in cognition and the psychology of language to establish hypotheses about the processes at work, and test them out empirically.

Before arguing and deriving our specific hypotheses, what we do think happens is that the task, on a superficial level, is clear to all participants: The expat CEO wants an open discussion on business-related topics. The local managers, however, are cognitively unable to join this discussion in the way the CEO hopes. As this fact itself becomes clear, the reasons involved – the meta-cognitive explanations available to the participants – are not shared as a social reality. This situation is not caused simply by cultural “habit,” but by fundamental differences in the cognitive requirements for carrying out conversations in their native languages. Hoping to reduce the resulting awkwardness, the CEO instead exacerbates the situation by wanting to show that speaking up is safe. The locals may be cognitively barred from adopting this option and their awkwardness increases instead. Neither party have access to a meta-cognitive way out.

The effect may not simply be due to different cultural “values” or viewpoints on authority that could potentially have been subject to discussion. With this study, we want to addresses the possibility that cognitive and meta-cognitive skills differ underneath the superficial layer of a learnt second language between Chinese and Western managers working in the same organization. These groups are interesting as examples of wide differences in linguistic and cultural heritage (Cavalli-Sforza, 2001), and the primary focus of this study is language as a carrier of culturally dependent behavior. Simultaneously, it raises the issue of culture in a wide range of ethnic, national, local, and corporate senses of the word. The concept of culture is itself too broad to be dealt with specifically here. However, before bringing up the concept of language and meta-cognition, it is important to bear in mind that the terms “Chinese” and “Western” are themselves broad. There are certainly many cultural differences within China, just as the word “Western” may encompass Anglo-Saxon, Germanic or Latin cultures, to name a few.

The literature below builds partly on studies of organizational behavior related to multinational companies operating in the growing Chinese economy during the recent decades. Also, partly, it builds on studies in linguistics and cognitive psychology where there has emerged initiatives to make the predominantly Anglo-Saxon research output less dominant (Henrich et al., 2010). In this tradition, there has been a tendency to group cognitive phenomena as much according to linguistic families of the subjects than to national boundaries, such as “indo-european,” “sino-tibetan,” etc (Renfrew, 1987; Cavalli-Sforza, 2001; Nisbett et al., 2001; Boroditsky, 2011; Mehler et al., 2011).

In what follows, the review of the research literature will use the original labels of the research, sometimes referring to nations and at other times using broader terms such as “Western” or “East Asian.” The use of these labels does not imply the assumption of cultural identity between (or even within) Asian nations, but simply indicates the concepts as used in the original research.

Attempts at untangling the relationship between thought and language in culture have been undertaken since antiquity, as people have wondered about the link between the obvious cultural and linguistic differences with strangers. The linguistic relativity hypothesis holds that language somehow shapes and limits thought and that accurate translation between two languages will be impossible. The opposite position is that language simply offers a “nomenclature” for thought that differs among languages, but the link between cognition and external reality remains independent of language (Gumperz and Levinson, 1996). In this case, knowledge of two languages would open for an exhaustive translation of meaning between the two if at least one speaker has sufficient command of both.

The most extensively formulated viewpoint in favor of linguistic relativity hypothesis in modern science was the Sapir-Whorf-hypothesis (Gumperz and Levinson, 1996; Pavlenko, 2016). To quote Whorf: “Language is not merely a reproducing instrument for voicing ideas but rather is itself the shaper of ideas, the program and guide for the individual’ s mental activity” (Whorf, 1956, p. 212).

While being seen as refuted in the 1970s, later research has created a more complicated picture. Theoretical improvements in the understanding of cognition, language and speech acts with improved experimental techniques now suggest that language learning is a likely and powerful gateway to the “cultural” programming of our minds (Gumperz and Levinson, 1996; Lucy, 1996; Fausey et al., 2010; Boroditsky, 2011; Pavlenko, 2016; Regier and Xu, 2017).

Language has been shown to shape the way humans organize as fundamental physical experiences as spatial relationships among objects (Bowerman, 1996), and differences in the use of bodily or absolute references in spatial references as well as agency in action (Papafragou et al., 2002, 2006, 2007; Boroditsky and Gaby, 2010). It is obvious that language changes how people attend to and re-construct narratives from visual storylines (Slobin, 1996), construct and remember agency (Fausey et al., 2010), differentiate emotions (Perlovsky, 2009), and grammatical constructions in languages will affect the way the physical environment is attended to (Lucy, 1996).

The original Sapir-Whorf hypothesis also proposed linguistic determinism, i.e., that language also restricts experience so that language also defines the borders of what may be experienced. By today’s knowledge, this may not be strictly true (Whorf, 1956; Pinker, 1994; Chandler, 1995; Gumperz and Levinson, 1996). But for reasons relevant to the present study, the communicative requirements of languages will shape the habits and guide the attention of people in different ways during dialogues.

First, the semiotic meaning of words and grammar is underspecified. The meaning of utterances is far less determined than most people think, opening for a constant need of contextualization. The ability to arrive at common understanding in language requires extensive knowledge not only about culture, but also about the way speech acts are used in a community to accomplish common intentions or “deixis” (Gumperz, 1996; Hanks, 1996). The degree of indeterminacy in languages is also varying. While Germanic languages such as English can be described as low-context languages, Chinese is a high-context language requiring more understanding of context and the relationships among speakers to establish deixis (Hall, 1977).

Second, these speech act practices probably shape grammatical structures throughout history. The pervasive structural differences between languages may be understood as habitual grammaticalizations of routine communicative practices. To quote Hanks (1996, p. 266): “Rather than asking what speakers of a given language *can* think because of the categories of their language, the question is what they routinely *do* think, because of the contours of their practices.” Routine cognitive operations such as those performed in using grammar and other schemata become automatized as pattern-recognizing mindsets, guiding attention and releasing automatic cognitive structures (Gollwitzer, 1990; Bayer et al., 2009).

Whether language shapes thought or culturally engrained cognitive patterns shape language is then a moot point, as speakers of any language need to “speak to think… [language] directs us to attend – while speaking – to the dimensions of experience that are enshrined in grammatical categories” (Slobin, 1996, p. 71). A wide range of habitual cognitive differences have been found between East Asians and Western subjects (Nisbett et al., 2001; Nisbett, 2003), although some of the findings may be dependent on context and task (Imai et al., 2010). One pervasive difference may be that Westerners prefer an analytic perspective in composing perceptions, while East Asians seem to prefer a holistic approach, attending more to the relationship among the elements in the percepts. This is also in accordance with differences in social exchange in general, where Westerners are rule-based in their expectations of social events, while East Asians tend to be relationship-oriented (Li et al., 2004).

A linguistic concomitant of this is a tendency for Westerners to group words according to semantic rules whereas Chinese are more likely to group words according to thematic relations (Ji et al., 2004). In the present study, we will be using this tendency as an indicator of the degree to which subjects are applying a culturally specific mode of language and cognition, thus Hypothesis 1:

*In a lexical classification task, native speakers of English will be more inclined to group nouns based on their semantic relationships, whereas native speakers of Chinese will be more likely to use relationship-based criteria.*

This habitual difference in verbal attention may affect mutual understand in verbal exchange between Westerners and Chinese. Mutual understanding (“deixis”) requires the establishment of a more thorough here-and-now than just space and time (Senft, 2014). Outlining what she calls an “indexicality principle,” Ochs (1996, p. 410) lists the following additional dimension: social identity (including relationships among speakers), social acts (the intended evocation of behavior), activity, affective stance and epistemic stance. Challenges emerging from different dialogue patterns among Chinese and Westerners is frequently attributed to power differences (Hofstede, 2001; Hofstede et al., 2010), although Chen and Tjosvold (2006) showed that the use of power in China may sometimes actually support speaking up. We instead propose a complementary explanation related to different requirements for deixis (Cresti, 2016), a phenomenon that appears to be influenced by linguistic relativity and particularly so along the East-West cultural dimension (Choi and Bowerman, 1991; Kashima and Kashima, 1998; Boulin, 2017). If native speakers of English are more free to infer from semantic classification than their Chinese counterparts (cf. Imai et al., 2010) the English native speakers can establish deixis by complying with the request is to engage in talk about the situation in question. Native speakers of Chinese, however, may need to establish deixis by attending to their superior’s intention.

According to the “semantic triangle” of Richards and Ogden (1989), words exist as spoken symbols (e.g., the word “brainstorm”), as a reference to actual instances (an actual “brainstorm”) and as an intention (whatever the speaker may mean by “brainstorm”). The semantic determinism that stems from semantic classifications may allow speakers more freedom to attend to the “referent” or topic matter, making deixis easier to attain. In the case where knowledge about the speaker’s intentions is crucial, participants in the conversation may need more knowledge about the speaker’s intention to establish deixis. This would be in line with the classification of languages and cultures as high vs. low context (Hall, 1977), where the Chinese language has developed as a high-context language requiring high degree of shared context to establish deixis (Fei et al., 1992). Being relationship rather than rule- or semantically oriented, Chinese managers will be more likely to remain quiet until they have a clearer picture of their leader’s intention. We therefore formulate Hypothesis 2:

*When judging a request by an English-speaking manager for his Chinese management team members to speak freely, native speakers of English will tend to guess that the Chinese managers will speak whereas native speakers of Chinese will be more inclined to guess that the Chinese management team will remain silent.*

Language being the most likely vehicle of social cognitive socialization (Ochs, 1996; Fausey et al., 2010), it turns out that speakers of a second language tend to re-create the cognitive constructions from their own native language, along with the most prevalent types of speech-acts in their cultural background, and these are also the aspects of language that are most resistant to change (Gumperz, 1996; Hanks, 1996). However, language socialization is a life-long and ongoing process that also benefits speakers of second and third languages (Ochs, 1996; Boroditsky, 2001; Pavlenko, 2016) and it is likely that language training will affect the ability to predict outcomes of cross-cultural speech activities through multi-language awareness, such as when managers with different native languages have problems establishing common understanding. We therefore formulate Hypothesis 3a:

*Asked to choose the most likely outcome of communication problems between an English-speaking CEO and his/her Chinese-speaking top management team, the likelihood of guessing right will be significantly predicted by proficiency in Chinese language.*

Conversely, the cognitive socialization of learning to speak English may make Chinese more likely to assume that English-speaking managers will go along with the linguistic habits of that language, i.e., speaking up. Existing research has shown that bilinguals will adopt different cognitive judgments depending on the language they are using to solve a task (Ji et al., 2004; Lee et al., 2010). It is therefore interesting to see whether English-speaking Chinese are less likely to predict a traditional Chinese response, thus Hypothesis 3b:

*Asked to choose the most likely outcome of communication problems between an English-speaking CEO and his/her Chinese-speaking top management team, Chinese respondents with proficiency in English language will be less likely to predict a traditional Chinese response than not so proficient speakers.*

Meta-cognition is thinking about thinking, monitoring and adjusting one’s own thinking while learning new skills (Triandis, 1995), which may also be useful in understanding communication skills in cross-cultural management (Mor et al., 2013). This has been shown to emerge as a consequence of increasing competence in a field rather than being a constant aspect of personalities (Kruger and Dunning, 1999; Ehrlinger et al., 2008). We would therefore expect bilingual individuals to have a more pronounced understanding of why communication may fail between participants from different cultures, as learning more languages has been shown to increase multi-language awareness (Pavlenko, 2016). We therefore formulate Hypothesis 4:

*Subjects speaking two languages will more often be able to give explanations similar to their foreign counterparts for why communication problems occur in the interaction between an expat CEO and a local management team with different linguistic background.*

If frequent mental operations may create automatic cognitive mindsets (Gollwitzer, 1990; Freitas et al., 2004; Gilbert et al., 2009), then these may influence the meta-cognitive explanations for speech-acts. In interactions between Westerners and East Asians, the meta-cognitive models may be related to the strength of the individual’s inclination toward semantic lexical categorization in English. Conversely, learning a high-context language such as Chinese requires a continuous vigilance directed at the intentional status of interlocutors that could also give rise to concomitant meta-cognitions. Thus Hypothesis 5:

*Subjects’ tendency toward semantic categorization of words is positively related to Western style meta-cognition and negatively related to Chinese-style meta-cognition.*

The belief whether the management team will speak up or not may simply be based on personal experiences in episodic memory. This expectation may, however, also be based on the linguistic habits of the speaker. In its strongest form, one could argue that semantic categorization promotes a world view where agentic verbal behavior is less restricted than in a thematic-relational linguistic community. And so our final Hypothesis 6 states:

*A preference for semantic over thematic-relational categorization will make it more likely that respondents believe that the Chinese management team will speak up when invited to a brain storm.*

## Method

This study was designed to avoid the possibility of culture blindness that has been shown to be prevalent in studies using Likert-scale measurement instruments (Kamnerdmongkhol and Bjørnstadjordet, 2019; Arnulf and Larsen, 2020; Casas Klett and Arnulf, 2020). To ensure sensitivity to the cultural differences in focus, the design is experimental in nature. Respondents are presented with a scenario and classification tasks, and some options for meta-cognitive explanations are offered. Scoring items with semantic relationships are intentionally avoided, along with the need to establish traditional measures of alpha reliabilities (for details, see Arnulf et al., 2018; Arnulf and Larsen, 2021).

### Sample

A total number of 196 participants were recruited and surveyed through the internet on two university campuses. About half of these were Chinese nationals with Chinese as their mother tongue. Some, but not all of these had learnt and practiced English since their school years. Similarly, the group of people labeled as “Westerners” were foreigners from Europe or North America who worked or studied in China. In this group, all participants had either English or another indo-european language as their mother tongue. Some, but not all, had learnt Chinese to varying degrees later in life. This sample was divided into four groups along two dimensions according to nationality of the participants and the language they used to answer the questionnaire (see Table 1). Thereby, the two ethnicities are mirror images of each other in terms of linguistic proficiency.

**TABLE 1:** Sample language characteristics and grouping.

	Ethnicity				
	Chinese (*N* = 100)		Western (*N* = 96)		Totals
		
	Male	Female	Male	Female	
Chinese language survey	26	25	24	22	97
English language survey	18	31	25	25	99

The data were collected 100% anonymously with no possibility of tracing any information back to individuals. Moreover, no questions were deemed sensitive, and all respondents were participating with the possibility to withdraw from the study at any time.

Group 1 consisted of 51 randomly recruited Chinese participants who were presented a Chinese language version of the questionnaire (26 males, 25 females). This group had some knowledge of English from school or work.

In Group 2, we selected 49 Chinese participants with Mandarin as native language and who have learnt English at school or at work. These were asked to answer the English language version of the questionnaire (18 males, 31 females).

Group 3 consisted of 50 Western participants living in China, who speak English but did not speak Mandarin. This group was asked to answer the English language version of questionnaire (even number of males and females).

Group 4 consisted of 46 Westerners who speak English and have learnt varying levels of Chinese. This group was asked to do the Chinese language version questionnaire (24 males, 22 females). At the time of data collection, Westerners with this proficiency in Chinese were still comparatively rare. We therefore obtained the assistance of language learning centers to access such respondents. This way of recruiting a sizable group of Western respondents with proficiency in Chinese skewed the age distribution to some extent, which will be addressed in the section “Results.”

We had no opportunity to administer formal tests of language proficiency. However, the two groups answering in a foreign language (Chinese responding in English and Westerners responding in Chinese) had to demonstrate a certain fluency in these languages to be able to complete our experimental condition. Since our interest concerns the effect of learning language at a mature age, we did not choose “true” or “compound” bilinguals (being brought up in a bilingual environment, cf. Ji et al., 2004).

The Chinese sample was slightly, but significantly, older than the sample of respondents with Western background. The Chinese sample also had a wider age range, see Table 2.

**TABLE 2:** Age distribution.

		15–20	21–25	26–30	31–40	41–50	51–60	>60 years
Ethnic background	Chinese	0	37	39	22	1	0	1
	Western	4	71	18	3	0	0	0

In terms of language proficiency, there is a small difference between the two groups. 93 respondents report that Chinese is their native language, but only 72 of the Westerners cite English as their native language, the rest as “advanced speakers” and two “intermediate.” No participant was truly bilingual with English and Chinese being their native language. More importantly, some of the Chinese respondents have some level of knowledge in English, but a substantial part of the Western respondents have absolutely no knowledge about Chinese, see Table 3.

**TABLE 3:** Language proficiency distribution.

		Chinese proficiency				
		No knowledge	Basic	Intermediate	Advanced	Native speaker
English proficiency	No knowledge	0	0	1	1	7
	Basic	0	1	2	2	31
	Intermediate	2	1	0	1	36
	Advanced	11	1	7	1	19
	Native speaker	29	4	13	26	0

### Procedures

A survey containing the four tasks described below was given to the respondents. Respondents who could only speak one language were responding to a survey version in their own mother tongue. Previous research has shown that bilinguals are more likely to change cognitive styles when being prompted in a different language (Lee et al., 2010). We therefore chose to ask respondents who were fluent in a second language to respond in their second language to maximize the probability of creating cognitive influence from this language.

Language proficiency was measured by one self-rating item. We had no objective measure, but the ability to read and answer the survey suggested that the proficiency of the bilingual groups were above “minimal.”

To test Hypothesis 1 concerning lexical categorizations, we chose a task previously described by Ji et al. (2004). The participants were asked to perform three categorization tasks where a target word (e.g., “carrot”) was presented along with three other words. Of these three words, one was taxonomic/semantic (“green pepper”), one was thematic/relational (“rabbit”) and one was simply irrelevant (“telephone”). The subjects are then asked to select one out of these three that they perceive as most closely related to the targeted word. We coded the responses as +1 for each taxonomic-semantic response, −1 for each relationship-oriented response and 0 for each irrelevant response. It was thus possible to have a categorization score from –3 (strong relational orientation) to +3 (strong semantic orientation).

### Testing of Hypotheses 2 and 3

To test the subjects’ ability to predict the outcome of a cross-cultural managerial challenge, we presented our participants with a short, unfinished scenario. The scenario is a story about a European coming as expatriate manager to China for work. Filling the position of the local CEO, he calls a top management team meeting where he asks his Chinese top management to feel free to present him with their view of the company’s local challenges, and invites them to bring in their opinions. The survey provided the subjects with several options for what they think would be most likely to happen, from an energetic group discussion to total silence. The scenario has been used courses on cross-cultural leadership by one of the authors over the years 2008–2020 and it is rated as “very realistic” by an independent sample of 610 experienced Chinese and expatriate managers in China (rated on a scale from 1 to 7, the mode is 7, the median is 6, and the standard deviation is 1.36). These statistics have not changed significantly over the years despite the substantial experiences that have accumulated in the interface between Chinese and global business. The stability of this situation testifies to the pervasiveness of the communicational divide, anchored in culturally determined cognition.

### Testing of Hypotheses 4 and 5

To examine the respondents’ meta-cognition, we asked participants to rate the likelihood of various explanations for their choice of predictions in the scenario. These were questions about why they thought the Chinese managers would participate in the discussion actively or keep quiet on a Likert scale from 1 (“least likely to be the reason”) to 7 (“most likely to be the reason”). We only asked questions about the ending that the subjects themselves believed most likely, i.e., we did not introduce or ask about explanations for the other possibility.

## Results

Table 4 shows the Spearman rank-order correlations among the key variables. The lexical categorization is strongly correlated with nationality and language proficiency, as hypothesized.

**TABLE 4:** Spearman rank-order correlations among key variables.

	1	2	3	4	5	6
1. Gender						
2. Age	0.06					
3. Chinese proficiency	0.05	0.30^**^				
4. English proficiency	0.02	−0.42^**^	−0.60^**^			
5. Test language	0.08	−0.20^**^	−0.39^**^	0.04		
6. Ethnicity	−0.07	−0.43^**^	−0.78^**^	0.82^**^	0.03	
7. High semantic category	0.02	−0.29^**^	−0.68^**^	0.56^**^	0.31^**^	0.66^**^

^**^ Correlation is significant at the 0.01 level (2-tailed).

The effect of demographics, language proficiency and ethnicity on semantic classification were explored in hierarchical regression (see Table 5). Controlling for age and gender, the most important predictors of semantic classification were language proficiency in Chinese and English. When ethnic background was entered, only the effect of proficiency in Chinese was significant, but the effect of English proficiency and ethnicity disappeared. This indicates that language proficiency was an important predictor of lexical classification. Chinese respondents answering in English were significantly more likely to use semantic classification than their non-English-speaking compatriots (mean difference 0.68 points, *p* = 0.05), but there was no difference among the Westerners answering in Chinese or English (both groups more likely than Chinese to make lexical judgments). Hypothesis 1 was therefore supported.

**TABLE 5:** Prediction of semantic lexical classification in hierarchical regression.

Dependent variable: Degree of semantic categorization.			
	Model 1	Model 2	Model 3
	**Adj. *R*^2^:0.09**	**Adj. *R*^2^:0.50**	**Adj. *R*^2^:0.51**
**Independent**	**Standardized β**		

Gender	0.04		
Age	−0.30**		
	**Independent**	**Standardized β**	
	Gender	0.03	
	Age	−0.03	
	Chinese proficiency	−0.53**	
	English proficiency	0.25**	
		**Independent**	**Standardized β**
		Gender	0.04
		Age	−0.2
		Chinese proficiency	−0.45**
		English proficiency	0.16
		Ethnicity	0.17

In an attempt at untangling the effects of learned language on cognition and predicted responses, we investigated the effects of ethnicity, language proficiency and the propensity to do semantic lexical classification on the respondents’ ability to guess that the Chinese managers would fall silent when asked to speak up. A two-by-two table plotting ethnicity against the respondents’ expectations of scenario outcomes showed that Chinese nationals are far more likely to predict a silent response than Westerners (χ^2^ = 10.10, *df* = 1, *p* < 0.01), thus supporting H2.

A similar table based on five levels of English proficiency shows an even stronger tendency (χ^2^ = 16.21, *df* = 4, *p* < 0.01), similar to Chinese proficiency (χ^2^ = 21.21, *df* = 4, *p* < 0.01). Conversely, a preference for semantic lexical classification significantly predicted the respondents’ prediction of whether the managers would speak up or be silent (binary logistic regression, Nagelkerke *R*^2^ = 0.06, Exp(B)0.84, *p* < 0.01). When entering lexical classification score, Chinese and English language proficiency and ethnical background as variables, the model becomes even more predictive (Nagelkerke *R*^2^ = 0.13, percentage correct guesses: 68.4, *p* < 0.01). Doing this in hierarchical regression singles out proficiency in Chinese language as the most predictive variable, rendering ethnicity insignificant.

**FIGURE 1:**
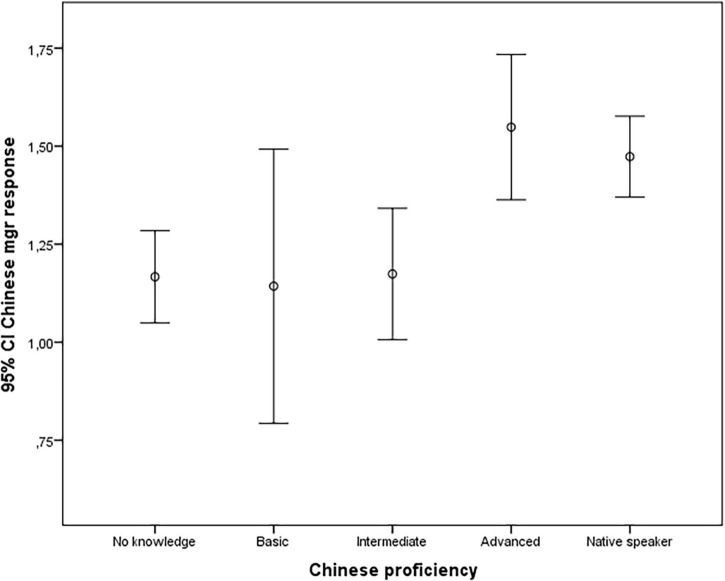
The relationship between proficiency in Chinese language and the tendency to guess the most likely outcome of the scenario.

**FIGURE 2:**
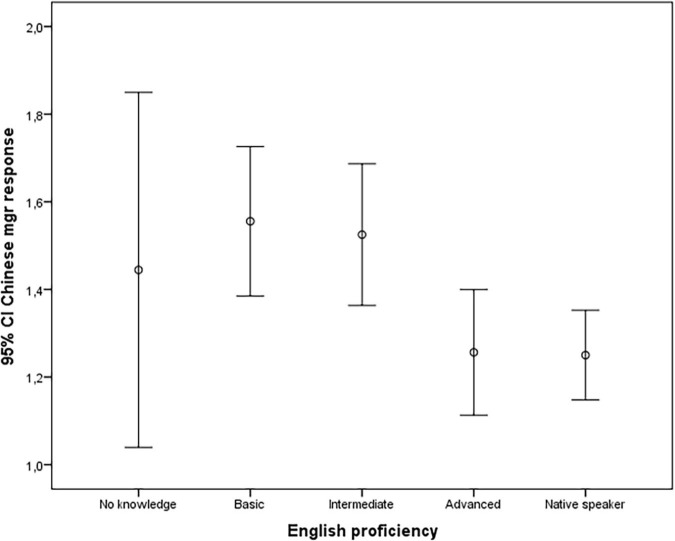
The relationship between proficiency in English language and the tendency to guess the most likely outcome of the scenario.

Thus, Hypotheses 3a and 3b were supported. Figures 1, 2 also indicate that a minimum level of language training may not be enough. Only advanced speakers of a foreign language are significantly more likely to predict a possible response that deviates from their own cultural norm.

To test Hypothesis 4, we first checked whether Chinese nationals would align their explanations with those of Westerners more with increasing proficiency in English when predicting that the Chinese managers would speak up in the discussion. This did not seem to be the case – there was no detectable and significant shift of explanations given related to the level of English proficiency. We repeated this for Westerners, but neither in this case did we find any significant shift in meta-cognitive comments related to level of proficiency in Chinese. Hypothesis 4 was therefore not supported in that we found no significant changes in meta-cognition based on language proficiency.

We explored this more in detail by comparing first the meta-cognitive reasons given for the managers’ silence by Chinese and Western respondents. This is shown in Table 6.

**TABLE 6:** Reasons cited for why the managers keep quiet compared between the two ethnic groups.

Explanation given for the Chinese managers’ silence in the meeting	Mean diff. Chinese-Westerners	Sign. level	
How realistic is this scenario?	0.04	n.s.	
The Chinese managers are afraid that they are not expressing the answer expected by CEO.	1.72	0.000	Chinese score higher
The other Chinese managers will suspect the motivation	1.22	0.003	Chinese score higher
The Chinese managers suspect the new CEO’s motivation for this brainstorm meeting.	1.10	0.012	Chinese score higher
The Chinese managers are afraid that their idea will not be recognized by the rest.	0.94	0.018	Chinese score higher
The Chinese managers feel that they may take risks by expressing their ideas and opinions.	0.66	0.054	Chinese score higher
It is the Chinese culture that one had better to wait for leaders or CEO’ s ideas or opinions	0.54	n.s.	Chinese score higher
The Chinese managers are afraid of losing faces.	0.15	n.s.	Chinese score higher
It is the Chinese culture that one had better not express ideas in the brainstorm meeting.	0.01	n.s.	Chinese score higher
The Chinese managers are influenced by their previous leaders’ style.	0.00	n.s.	Chinese score higher
The Chinese managers are afraid that the CEO loses his face.	−0.88	n.s.	Westerners score higher

The Chinese respondents are largely in favor of three explanations: The managers simply do not know what the CEO wants to hear, they are afraid of the reactions from their fellow managers, and they think they should wait for their boss to speak up before having their own opinions. This explanation is in line with the concerns about deixis stated earlier – the communicative situation is too ambiguous and Chinese managers may simply not have a clear alternative without risk for conflicts or internal strife. This does not seem obvious to the Westerners, who believe the situation is mostly about saving face. “Saving face” is a more superficial explanation that does not seem to be of the same concern to native Chinese.

We then compared all reasons given to see if there were differences or similarities in reasons among Chinese and Westerners who believed that the managers would speak up, and also compared this to the responses from the other scenario on the same explanations (see Table 7).

**TABLE 7:** Comparison of meta-cognitive option rankings for Chinese and Western respondents by their choice of predicted course of events.

	Mean score				
	Predicting silence		Predicting speaking		
	Chinese	Western	Chinese	Western	
The Chinese managers are afraid that the CEO loses his face if they do not participate actively.	3.44	4.32	4.25	3.20	The Chinese managers are afraid that the CEO loses his face if they do not participate actively.
The Chinese managers feel that they may take risks by expressing their ideas and opinions.	5.58	4.92	4.19	5.17	The Chinese managers feel comfortable to express their ideas.
It is the Chinese culture that one had better not express ideas in the brainstorm meeting.	4.81	4.83	3.67	3.31	It is the Chinese culture that one had better not express ideas in the brainstorm meeting.
The Chinese managers suspect the new CEO’s motivation of this brainstorm meeting.	4.58	3.48	3.67	5.16	The Chinese managers believe the there are no other intentions behind this task.
The other Chinese managers will suspect the motivation of others	4.98	3.76	4.92	3.83	The Chinese managers feel they can be more a competitive than the rest.

Looking at the explanations for why the Chinese managers would choose to speak, there are again differences between Chinese and Westerners. The Westerners seem to believe that the Chinese managers trust the new CEO and feel comfortable speaking out. This does not seem convincing to Chinese respondents, not even to those who are in favor of the speaking. These Chinese respondents are more skeptical toward the new CEO’s intentions and the risks involved, as their compatriots who predict silence. When they choose to predict that the managers will speak out, it seems to be partly because they think they will please the CEO by doing so, and because they see it as a legitimate arena for competition among the members of the top management team. However, the Chinese respondents still seem to assume that risk is involved, as contrary to the Westerners who see speaking out as a comfortable arena for exercising verbal agency.

Testing Hypothesis 5, Spearman-correlations were calculated for the relationships between proficiency in English, Chinese and the tendency toward semantic lexical categorization. As can be seen from Table 8, there is an almost consequent inverse relationship between metacognition and level of proficiency in the two languages. In a regression equation, semantic lexical classification significantly and positively predicts English proficiency (adj *R*^2^ = 0.33, β = 0.58, *p* < 0.01) and is significantly and negatively predicting proficiency in Chinese (adj *R*^2^ = 0.46, β = −0.68, *p* < 0.01). Hypothesis 5 was therefore supported.

**TABLE 8:** Spearman correlations for meta-cognitive explanations with proficiency in English, Chinese, and semantic lexical categorization, by predicted outcome of the scenario.

Meta-cognitive reasons for keeping silence in the meeting (*N* = 73)	Chinese profic.	English profic.	High semantic category	Perceived realism
The Chinese managers are afraid of losing faces.	0.07	−0.11	−0.01	0.11
The Chinese managers are afraid that the CEO loses his face.	−0.12	0.25^*^	0.17	0.17
The Chinese managers are afraid that they are not expressing the answer expected by CEO.	0.49^**^	−0.33^**^	−0.12	0.21
The other Chinese managers will suspect the motivation	0.43^**^	−0.27^*^	−0.14	0.17
The Chinese managers feel that they may take risks by expressing their ideas and opinions.	0.23^*^	−0.28^*^	0.01	0.36^**^
The Chinese managers do not know which opportunities and challenges the China branch	−0.07	−0.15	−0.24^*^	−0.15
It is the Chinese culture that one had better not to express ideas in the brainstorm meeting.	−0.03	−0.13	−0.15	0.10
It is the Chinese culture that one had better to wait for leaders or CEO’ s ideas or opinions	0.13	−0.16	−0.12	0.36^**^
The Chinese managers are afraid that their idea will not be recognized by the rest.	0.29^*^	−0.37^**^	−0.22	0.04
The Chinese managers feel that they are not the proper person to answer the question. The senior managers should answer first.	0.39^**^	−0.31^**^	−0.05	0.16
The Chinese managers suspect the new CEO’s motivation for this brainstorm meeting.	0.23^*^	−0.20	−0.08	0.05
The Chinese managers are influenced by their previous leaders’ style.	−0.10	−0.08	0.08	−0.04
Do not hold such brainstorm meeting until mutual trust is built.	−0.09	0.08	0.11	−0.10
Talk to the Chinese managers one by one, instead of a brainstorm meeting.	0.05	0.09	−0.02	0.09
Obtain information through informal channels, instead of a formal meeting.	−0.03	0.09	−0.01	0.07
Accept and adapt to this situation, and avoid formal meetings in the future.	0.06	−0.19	−0.35^**^	−0.12
Know this situation, and try to make Chinese employees more involved and active in the future.	−0.03	−0.13	0.24^*^	−0.01

**Meta-cognitive reasons for speaking out in the meeting (*N* = 123)**	**Chinese profic.**	**English profic.**	**High semantic category**	**Perceived realism**

Call the roll to answer	0.32^**^	−0.37^**^	−0.12	−0.01
The Chinese managers are afraid that the CEO loses his face if they do not participate actively.	0.35^**^	−0.18	−0.27^**^	n.a.
The Chinese managers are afraid of blame from the CEO.	0.31^**^	−0.18^*^	−0.32^**^	n.a.
The Chinese managers are showing respect to the CEO.	0.26^**^	−0.13	−0.17	n.a.
The Chinese managers feel that they should participate in discussions like this.	−0.09	0.08	0.16	n.a.
The Chinese managers feel comfortable to express their ideas.	−0.36^**^	0.20^*^	0.29^**^	n.a.
It is the Chinese culture that one had better to express ideas in the brainstorm meeting.	0.13	−0.14	−0.17	n.a.
The Chinese managers want to satisfy the new CEO.	0.61^**^	−0.36^**^	−0.56^**^	n.a.
The Chinese managers believe the there is no other intentions under this mission.	−0.43^**^	0.39^**^	0.36^**^	n.a.
The Chinese managers feel they can be more a competitive than the rest.	0.37^**^	−0.28^**^	−0.35^**^	n.a.

^*^ Correlation is significant at the 0.05 level (2-tailed).

^**^ Correlation is significant at the 0.01 level (2-tailed).

Subsequently, we ran two different regression models, one general linear model and a logistic regression model. In both cases, the dependent variable was the prediction of silence or talk in the Chinese management team. Age and sex were control variables. Proficiency in the two languages were entered together with semantic classification preference and the experimental conditions (Chinese and Westerners in both language conditions). Both models predicted choice of response significantly (Nagelkerke *R*^2^ = 0.33 in binary regression, adjusted *R*^2^ for the GLM was 0.38), and in both models, only semantic preference alone and the interaction term with experimental conditions were independently significant. Hypothesis 6 was therefore supported.

Finally, since the Westerners were significantly different from the Chinese sample in age, there was a possibility that either age or its related variable work experience could have inflated the statistical differences. To check this possibility, we explored the correlation between age and endorsement of meta-cognitive explanations. While there were some weak but significant correlations there, these disappeared completely when controlling for linguistic proficiency in the two languages. It seems warranted to conclude that age did not play any substantive role in the reported statistics.

## Discussion

The purpose of this study was to explore the influence of two widely different languages (English and Chinese) on mono- and bilingual subjects’ ability to predict and explain a commonly occurring communication barrier in management involving Chinese and Western participants as earlier described in research (Smith et al., 1997; Chen and Tjosvold, 2005, 2006, 2007; Wilkinson et al., 2005; Arnulf, 2014; Arnulf and Kristoffersen, 2014; Casas Klett and Arnulf, 2020). In particular, the focus of the study was on how cognitive and meta-cognitive characteristics of the languages may obstruct the dialogue process while at the same time making it difficult for the organizational participants to overcome the communication barrier.

Research on cultural differences in cognition (Nisbett et al., 2001; Nisbett, 2003; Ji et al., 2004; Henrich et al., 2010) and re-formulations of the linguistic relativity hypothesis (Gumperz and Levinson, 1996; Fausey et al., 2010; Imai et al., 2010; Boroditsky, 2011; Pavlenko, 2016) were reviewed to suggest an explanation for the communicative challenges emerging from the situation. Speakers of low-context languages such as English may support their communication on semantic classifications relatively independent of the relationships among the speakers. Speakers of a high-context language such as Chinese will need to pay much more attention to the intentions of speakers to establish common deixis (Hall, 1977; Hanks, 1996; Cresti, 2016), thus needing to take a more complicated view of speech acts in to consideration to avoid misunderstandings (Hall, 1977; Gumperz, 1996; Ochs, 1996), probably also evoking cultural dependent patterns of authority such as power distance (Hofstede et al., 2010).

While the observed responses to the scenario may be rooted in diverse cultural, political and socio-economic factors influencing the participants, most hypothesized effects were supported by the empirical data: Hypothesis one was supported in that proficiency in English was strongly related to the tendency to perform logical-semantic classification of words in the lexical classification task. Proficiency in Chinese was conversely predictive of thematic-relational lexical classification. Asked to predict the outcome of a scenario from a top management team meeting, Chinese nationals were far more likely to predict an ensuing silence than Western nationals, who would expect the Chinese managers to speak up at their CEO’s request, thereby supporting Hypothesis 2. A closer inspection of their language proficiency showed that proficiency in either language influenced the prediction in the scenario. Westerners proficient in Chinese language were more likely to predict the silence in Chinese managers, and Chinese respondents proficient in English were more likely to assume that the Chinese managers in the scenario would comply with the request to speak out. Hypotheses 3a and 3b were thereby supported.

Hypothesis 4 suggested that with increasing proficiency in a second language, this should influence the meta-cognitive explanations for why the respondents’ choice of predictions in the scenario. This hypothesis was not supported. This finding is of particular interest to the phenomenon studied here: While studying a foreign language may create what some have called a “multilingual awareness” (Pavlenko, 2016), it takes quite advanced language learning to acquire the meta-cognitive skills to understand why the communicational problem unfolds in this way. In other words, complete cross-cultural literacy requires more than simply learning a language and acknowledging different cultural habits. There may be underlying cognitive structures that emerge with different native languages that also influence the interpretation of obstacles in communication.

A closer inspection of the respondents’ ratings of possible explanations showed notable differences among Chinese and Westerners’ reasons for predicting even the same outcomes of the scenario. Westerners were more likely to assume that the Chinese managers were restricted by concerns for keeping face, and that they could somehow become comfortable in speaking out, trusting their CEO. Comparatively, the Chinese respondents endorsing both predictions kept emphasizing a strong concern for the CEO’s unknown intentions, the risk for strife among the management team members and the power distance suggesting that the CEO should state his position first.

Hypothesis 5 proposed that the respondents’ lexical classification style would be strongly related to their metacognitive views. This hypothesis was also supported. A tendency toward lexical classification was strongly positively correlated with the pattern of meta-cognition typical of people proficient in English, whereas lexical classification was significantly and almost equally negatively related to the meta-cognitions of respondents proficient in Chinese. The wider implication of this is that the cognitive operations provided for by a native language seems to predispose speakers to meta-cognitive operations that differ substantially from speakers of another language. These differences in meta-cognition remain unaffected when sentences are translated from one language to another. Consequently, the speakers *speak* like their foreign conversational partners, but keep *thinking* like their fellow nationals.

And finally, the strongest hypothesis concerning semantic classification was supported – the tendency to choose semantic over thematic-relational classification also predicted the respondents’ assumptions of how the Chinese management team would react. Respondents in favor of semantic classification are more likely to assume that Chinese managers will speak up when asked.

On the one hand, these findings are in line with previous research on East-West differences in cognition and cross-cultural management communication (Chen and Tjosvold, 2013; Lin et al., 2018; Casas Klett and Arnulf, 2020). The differences in lexical classifications were similar to previous studies (Nisbett, 2003; Ji et al., 2004), and the different patterns in group communication among managers have been commented earlier (Wilkinson et al., 2005; Chen and Tjosvold, 2006). On the other hand, this study digs deeper into why and how linguistic differences may convey communicational barriers that are not easily crossed even by learning a second language. As shown by linguistic research, the meaning of speech acts are underspecified by the formal semantic and syntactic frameworks of languages, even low-context languages such as the Germanic languages (Hanks, 1996). Grammar can be seen as automatized conventions of social practices of speech acts, helping to tune speakers of a common language to the key features of situations that they need to communicate effectively (Slobin, 1996). While the cognitive structures of different cultures are not deterministic in the strictest sense of the Sapir-Whorf-hypothesis (Gumperz and Levinson, 1996; Ji et al., 2004; Imai et al., 2010; Pavlenko, 2016), it is precisely the grammatical features of a language with abstract, non-sensorical content that are most difficult to learn for non-native speakers (Hanks, 1996; Slobin, 1996). The socialization into different linguistic communities such as Chinese or English implies the routine habituation of widely different cognitive patterns with ramifications for social identity, social interaction, and epistemic stance (Gollwitzer, 1990; Bourdieu, 1996; Gumperz, 1996; Ochs, 1996).

Differences in behavior that come with differences in culture may have a wide range of roots, ranging from very local traditions through socio-economic, political, industrial and national cultures. It is definitely possible that some of the differences observed in this study may not be directly related to language and linguistic relativity. Still, the communicational obstacles are recognized as realistic by a wide community of practicing managers represented in this study. In line with Whorf’s original observations, the cultural differences take place in language and need to be sorted out in language (Whorf, 1956; Pavlenko, 2016).

Our findings suggest that even fluency in a second language may not bring about the meta-cognitions that explain and guide the speech acts of native speakers, in line with previous findings (Norenzayan et al., 2002; Harzing and Pudelko, 2014). While research on incompetence and meta-cognition shows that training a skill will increase meta-competence, our findings suggest that the mere ability to enter new linguistic community in a second language may still render the new speaker “unskilled and unaware of it” (Kruger and Dunning, 1999; Ehrlinger et al., 2008). This is similar to what linguists have recently called “false friend.” A “false friend” is a phrase or a speech act that sounds as intelligible to the speaker of a new language since it seems similar to something that works in their own language, but is in fact indicating something completely different to the native speakers (Enfield, 2007).

An intriguing but uncertain implication of our findings is that speakers of low-context languages may rely on lexical semantic networks that are relatively independent of the speakers, which can account for the relatively free verbal exchange characteristic of Western societies since antiquity (Nisbett et al., 2001). The ensuing pragmatic practices in speakers of these languages make it very difficult to empathize with speakers of languages with different origins, who will have a different approach to how speech acts relate to the co-ordination of actions and establishment of knowledge (Nonaka and von Krogh, 2009). We believe our study contributes by linking more specific cognitive and linguistic perspectives on management-relevant situations and behaviors that so far have been dominantly explained by classifications of cultures such as the use of Hofstede’s (1980) dimensions, as pioneered and called for in recent publications on multi-linguistic management (Harzing et al., 2013; Harzing and Pudelko, 2016; Casas Klett and Arnulf, 2020).

## Practical Implications

Our findings contribute to the understanding of severe and often unexpected difficulties emanating from cross-cultural management, where knowledge and organization depends on communication among people who have widely different interpretations even if sharing the same working language such as English (Hofstede et al., 2010). While a growing number of multi-national companies locate R&D centers to places in other countries than their corporate headquarters, many of these find that the costs are much higher than estimations (Porter and Rivkin, 2012) and that knowledge does not transport well across languages and cultures (Nonaka and von Krogh, 2009), even in the case of simple sourcing operations (Wilkinson et al., 2005). Since even bilinguals may underestimate the cognitive differences affecting managerial communication and decision-making, our study may be valuable in increasing our understanding of language pragmatics in cross-cultural management.

## Limitations and Implications for Further Research

This is a cross-sectional study using a random sample of respondents. The design of the study is mainly correlational and we cannot make any causal claims about lexical semantic classification, grammar habituation and the meta-cognitions of practicing managers. In particular, language proficiency was self-rated without any other checks than that Chinese with English proficiency answered surveys in English and Westerners conversely in Chinese. However, the lexical classification was an independent task prior to making predictions in the scenario. We do believe that the present design has some ecological validity since the findings mostly generalize to expatriate managers who struggle to cope with organizational processes in a foreign language. To make more specific psychological inferences about the linguistic relativity hypothesis *per se*, more objective measures of language proficiency as well as national and international managerial experience should be included in the experimental design. A longitudinal design taking language learning and work experience into consideration could possibly give more information about the development of meta-cognition and the introduction of more scenarios would increase the reliability of the findings.

## Author’s Note

Jan Ketil Arnulf has taught leadership development at Fudan University in Shanghai, China, for 10 years. Wanwen Dai received his Ph.D. from Nanjing University. His current research interests are Cross-cultural management in FIEs, including Marketing, human resource management, and organizational learning.

## Data Availability Statement

The raw data supporting the conclusions of this article will be made available by the authors, without undue reservation on request to the corresponding author.

## Ethics Statement

Ethical review and approval was not required for the study on human participants in accordance with the local legislation and institutional requirements. The patients/participants provided their written informed consent to participate in this study.

## Author Contributions

JA: designing study, collecting data, analyzing, and writing. WD: collecting data, analyzing, and writing. HL and ZN: collecting data and literature research. All authors contributed to the article and approved the submitted version.

## Conflict of Interest

The authors declare that the research was conducted in the absence of any commercial or financial relationships that could be construed as a potential conflict of interest.

## Publisher’s Note

All claims expressed in this article are solely those of the authors and do not necessarily represent those of their affiliated organizations, or those of the publisher, the editors and the reviewers. Any product that may be evaluated in this article, or claim that may be made by its manufacturer, is not guaranteed or endorsed by the publisher.
